# Numbsense of shape, texture, and objects after left parietal infarction: A case report

**DOI:** 10.1111/jnp.12229

**Published:** 2020-10-17

**Authors:** Keisuke Hanada, Kayoko Yokoi, Akinori Futamura, Yuji Kinoshita, Kazutaka Sakamoto, Kenjiro Ono, Kazumi Hirayama

**Affiliations:** ^1^ Department of Rehabilitation Suishokai Murata Hospital Osaka City, Osaka Japan; ^2^ Graduate School of Comprehensive Rehabilitation Osaka Prefecture University Habikino City, Osaka Japan; ^3^ Department of Occupational Therapy Graduate School of Health Sciences Yamagata Prefectural University of Health Sciences Yamagata City, Yamagata Japan; ^4^ Division of Neurology Department of Medicine Showa University School of Medicine Tokyo Japan; ^5^ Department of Psychiatry Aizu Medical Center Fukushima Medical University Aizuwakamatsu City, Fukushima Japan

**Keywords:** numbsense, covert recognition, cortical somatic sensation, secondary somatosensory cortex, dorsal stream

## Abstract

Numbsense is a phenomenon, wherein patients can correctly respond to somatosensory stimuli at a higher rate than expected by chance, but cannot perceive the same stimuli consciously. Previously, numbsense has been reported in tactile localization of stimuli on the patient’s own body. Here, we describe a patient with numbsense that involved touched objects. The patient could not recognize the majority of somatosensory stimuli after left parietal infarction, but could correctly select shape, texture, and object stimuli more frequently than expected by chance.

Numbsense is a phenomenon similar to blindsight in vision, wherein patients can correctly respond to somatosensory stimuli at a higher rate than expected by chance, but cannot perceive the same stimuli consciously (Paillard, Michel, & Stelmach, 1983; Rossetti, Rode, & Boisson, 1995). Paillard et al. (1983), who was the first to report this phenomenon, examined the somatosensory function of a patient who had a cerebral lesion and observed that even though the patient did not have the sense of touch, he could correctly localize the touched position on his body. Rossetti et al. (1995) also reported a patient with severe somatosensory dysfunction after a left thalamic infarction. Like the patient reported by Paillard et al., Rossetti et al.’s patient remained able to point out the location of the stimulus with an above‐chance level.

As described above, in previously reported patients, numbsense has been observed in tactile localization of stimuli on their own bodies. Lesions involved the primary somatosensory cortex (S1) and secondary somatosensory cortex (S2) (Paillard, 1983) or the ventroposterolateral nucleus of the thalamus (Rossetti et al., 1995). The mechanism for this type of numbsense has been regarded as a transmission of information through the medial portion of the posterior complex of the thalamus to the posterior parietal area, without passage through the S1 (Rossetti, Rode, & Boisson, 1995, 2001). Here, we describe a patient who could not consciously perceive stimuli of most primary and cortical somatosensory modalities after a brain infarction involving S2, but could correctly select shape, texture, and object stimuli more frequently than expected by chance. To the best of our knowledge, this is the first reported case of numbsense that solely involved touched objects.

## Case presentation

**Figure 1:**
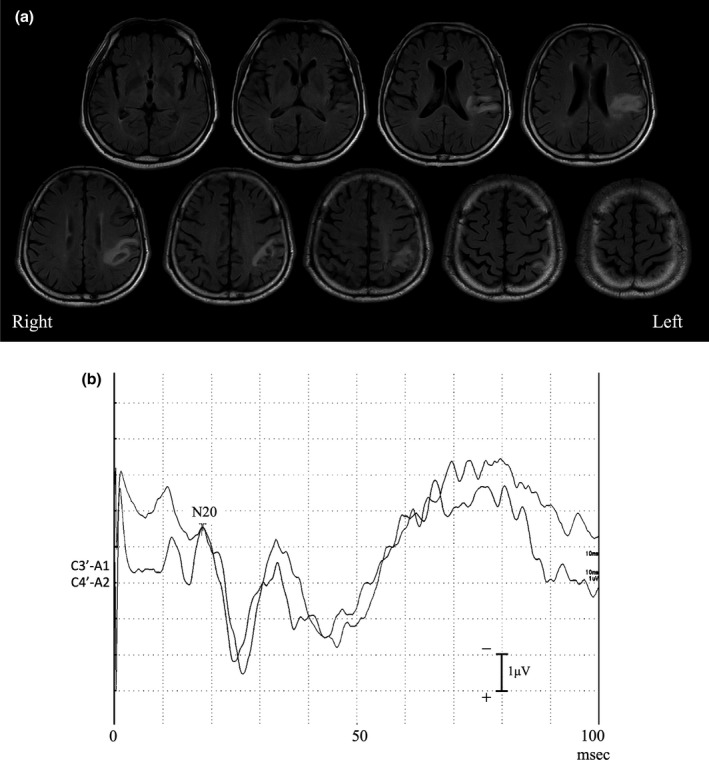
(a) Magnetic resonance fluid‐attenuated inversion recovery scan of the patient. Ischaemic changes were noted in the left lower postcentral gyrus, parietal operculum, upper posterior insula, and upper inferior parietal lobule. (b) Upper limb somatosensory‐evoked potentials one week after disease onset. N20 was evoked at 18.1 msec with 1.39 µV in the right hemisphere and at 18.1 msec with 1.29 µV in the left. C3’‐A1 (left hemisphere) and C4’‐A2 (right hemisphere).

A 76‐year‐old right‐handed man was admitted to our institution with acute stagger. He was a farmer with 12 years of school education. Magnetic resonance imaging (Figure 1‐a) showed infarctions in the left lower postcentral gyrus, parietal operculum, upper posterior insula, and upper inferior parietal lobule. Within 1 week of admission, the stagger had disappeared. Neurologically, the right side of the body showed severe somatosensory impairment, as described below. There were no other neurological abnormalities. We conducted the following neuropsychological examinations, somatosensory tests, and an examination of somatosensory‐evoked potentials. The patient provided written informed consent. The procedures complied with the ethical standards of the 1964 Declaration of Helsinki regarding the treatment of human participants in research.

## Methods

### Neuropsychological examinations

General cognition and intellectual ability were evaluated with a Mini‐Mental State Examination (MMSE) and the Wechsler Adult Intelligence Scale‐III (WAIS‐III). Language function was assessed by the Standard Language Test of Aphasia (SLTA) (Japan Society for Higher Brain Dysfunction, 2003) and the Token Test. General attention was tested with the Digit Span and Spatial Span tasks of the Wechsler Memory Scale‐Revised. Frontal‐executive function was assessed with Frontal Assessment Battery (FAB) and the Trail Making Test (TMT). Hemispatial neglect was examined with the Catherine Bergego Scale (CBS) and the Behavioral Inattention Test (BIT).

### Somatosensory testing

Each somatosensory function was classified into basic modality and cortical modality based on the dichotomy reported by Klingner and Witte (2018). Somatosensory tests were performed under shielding to ensure that the patient could not see them.

#### Basic somatosensory modalities

##### Touch

Tactile sensitivity over the centre of the palm and each fingertip was tested using the Semmes‐Weinstein aesthesiometer according to the procedure described by Bell‐Krotoski et al. (1995). The patient was also asked to immediately report when the examiner touched a portion of his upper limb with a short brush. The thenar, hypothenar, centre of the palm, anterior surface of the forearm, posterior surface of the forearm, and each of the fingertips were stimulated one time.

##### Pain

The patient was asked to immediately report when the examiner pricked a part of his upper limb with a pin. The thenar, hypothenar, centre of the palm, anterior surface of the forearm, posterior surface of the forearm, and each of the fingertips were stimulated three times.

##### Thermal

The patient was asked to indicate which cylinder was hotter after the consecutive placing two cylinders with different temperature (40°C and 10°C) on the palm of his hand. The thenar, centre of the palm, back of the hand, anterior surface of the forearm, and posterior surface of the forearm were stimulated, twice at each point.

##### Pressure

The patient was asked to immediately report when the examiner pressed on a part of his upper limb with a thin dull stick. The thenar, hypothenar, centre of the palm, anterior surface of the forearm, posterior surface of the forearm, and each of the fingertips were stimulated three times.

##### Position sense

The patient was asked to relax his hand and indicate whether his finger was flexed or extended when his finger was passively moved by the examiner. The distal interphalangeal joint of the index finger, distal interphalangeal joint of the little finger, and wrist joint were each moved 10 times by 50% of the normal range of motion.

##### Vibratory sense

The patient was asked to report whether a 128‐Hz tuning fork was vibrating when the examiner touched the patient with it. By gradually reducing the amplitude of the tuning fork, the patient was evaluated for left–right differences in the smallest detectable amplitude. The ulnar styloid and index PIP joints were stimulated.

Tests for touch, pain, thermal, pressure position sense, and vibratory sense were also conducted by two alternative ‘forced‐choice’ tests with the same methods as the detection task on different days, with the exception of vibratory sense.

#### Cortical somatosensory modalities

##### Size comparison

Square leather pieces with sides of 40, 45, 50, 55, 60, 65, and 70 mm were used. The patient was asked to consecutively place two pieces with different sizes in the palm of his hand and then advise which of the two stimuli was larger. We recorded the minimum size difference that the patient could detect with the above instrument. The patient was allowed to move his finger when putting these objects in his hand.

##### Weight comparison

Seven cylinders of the same size but different weights (10, 30, 50, 70, 90, 110, and 130 g) were used. The patient was asked to consecutively place two cylinders with different weights in the palm of his hand and advise which of the two stimuli was heavier. We recorded the minimum weight difference that the patient could detect with the above instrument. The patient was allowed to move his finger, but was not allowed to shake or drop the weight, when placing it in his hand.

##### Graphesthesia

The examiner wrote a digit on the patient's palm using a thin dull stick. The patient was asked to answer which digit was written. The number was allowed to be any Arabic digit (0 to 9).

##### Two‐point discrimination

The patient was asked to answer whether the stimulus given to the tip of the middle finger was one or two. The examiner recorded the minimal detectable distance of the two stimuli.

##### Tactile localization

The tips of the thumb, middle finger, and little finger, the thenar, hypothenar, anterior surface of the forearm, and the posterior surface of the forearm were stimulated with a thin dull stick. The patient was asked to touch the touched point using his contralateral limb.

##### Identification of three‐dimensional geometric figure

Six choices were always presented to the patient. All geometric figures were wooden, weighed almost the same (between 10 and 15 g), and had almost the same volume (between 18 and 23 cm^3^). The patient was first asked to verbally identify the figure on his palm and then forced to choose an answer from among six options as to what the touched stimulus was. The patient was asked to answer verbally what the figure on his palm was. The patient was allowed to move his finger, but was not allowed to shake or drop the figure, when put on hand. Spheres, cones, cylinders, cubes, triangle poles, and hexagonal cylinders were used.

##### Identification of materials

Six choices were always presented to the patient. The patient was first asked to verbally identify the material by actively touching it and then forced to choose an answer from among six options for what the touched stimulus was. Same size velcro, mesh, rubber, felt, file, and an aluminium plate fixed on the board were used for the test.

##### Identification of everyday objects

Six choices were always presented to the patient. The patient was first asked to verbally identify the object on his palm and then forced to choose an answer from among six options what the touched stimulus was. The patient was allowed to move his finger, but was not allowed to shake or drop the figure, when put on his hand. Keys, toothbrushes, shaver, comb, bottle openers, and clothespins were used.

The affected and non‐affected hands were tested the same number of times per session. The patient was examined seven times each for materials and geometric figures, and six times each for everyday objects. All trials for all tests were performed once, and it took four days to finish all the tests. We used a one‐sided Bayesian binomial test to verify whether the patient’s performance in testing for geometrical figures, texture, and everyday objects was above‐chance or not.

### Somatosensory‐evoked potentials (SEP)

SEP studies were carried out during the same week as the first clinical examination. Scores were recorded with an EP system (Neuropack S1 MEB‐9400 Nihon Kohden). Electrical stimuli were delivered by surface electrodes to the median nerve at the wrist. Stimulus duration was 200 microseconds, and intensity was adjusted to 1.1 times the motor threshold. The electrodes were placed contralateral to somatosensory areas (2 cm behind C3 and C4). An electrode at an earlobe was used as a reference. The USEP amplitude was measured over the hemisphere contralateral to the stimulated median nerve. Responses were filtered through a bandpass of 1 to 500 Hz. The average of 300 responses was used as the amplitude value.

## Results

### Neuropsychological observations

Neuropsychologically, the patient exhibited minimal conduction aphasia, deterioration of verbal short‐term memory, and acalculia, but had no problems with general attention, general cognition, episodic memory, or frontal function; he also showed no hemispatial neglect (Table 1).

**Table 1:** Results of neuropsychological tests

Test	Performance
Handedness	
Edinburgh handedness inventory (max: 100)	100
Language and calculation	
Standard language test of aphasia	
Listening (max: 100)	90
Speech (max: 100)	85
Reading (max: 100)	97.5
Writing (max: 100)	61.7
Calculation (max: 100)	60
Token test (max: 165)	139
General attention (short‐term memory)	
Digit span	
Forward	3
Backwards	3
Spatial span	
Forward	6
Backwards	6
General cognition	
Mini‐mental state examination (max: 30)	30
Wechsler adult intelligence scale‐III FIQ	108
Wechsler adult intelligence scale‐III VIQ	99
Wechsler adult intelligence scale‐III PIQ	119
Raven’s colored progressive matrices (max: 36)	32
Episodic memory	
Recall of three words (max: 3)	
Immediate	3
Post‐interference	3
He was able to give accurate oral descriptions of the contents of the previous day’s training.	
Frontal function	
Frontal assessment battery (max: 18)	18
Trail making test A (sec.)	95
Trail making test B (sec.)	143
Hemispatial neglect	
Catherine bergego scale (max: 30)	0
Behavioral inattention test (max: 146)	145

Note

max = maximum.

### Somatosensory ability

#### Basic somatosensory abilities

Basic somatosensory modality (Bell‐Krotoski et al., 1995; Klingner & Witte, 2018) testing (Table 2A) revealed loss of tactile, pain, thermal, and pressure sensations. The patient reported the absence of sensation during testing. When forced to select between the presence and absence of stimulation, the patient had remarkably few correct answers. When his position sense was tested, he was unable to notice the movement itself and could not decide whether it was upward or downward. The patient scored poorly in the 'forced‐choice' tests. The observed reduction in the patient’s sensation of vibration was slight, compared with that of the contralateral limb. The vibration sense of the right hand could be immediately detected if the amplitude was large, but the right hand could not detect the minimum amplitude that could be detected with the left hand.

**Table 2:** Results of somatosensory tests

Test	Performance	
	Left arm	Right arm
A. Basic somatosensory modalities		
Touch		
Semmes‐Weinstein Monofilaments (min:1.65, max: 6.65)	3.61	> 6.65
Touch with a brush (max: 30)		
Awareness	30	0
Forced choice (touched or not touched)	30	14
Pain		
Pinprick (max: 30)		
Awareness	30	0
Forced choice (pricked or not pricked)	30	15
Thermal		
Hot water (max: 10)		
Awareness	10	0
Forced choice (hot or cold)	10	3
Pressure (using a thin, dull stick; max: 30)		
Awareness	30	0
Forced choice (pushed or not pushed)	30	13
Position sense (max: 30)		
Awareness	30	0
Forced choice (upward or downward)	30	12
Vibratory sense	Normal	Slightly reduced
B. Cortical somatosensory modalities		
Size comparison (threshold, mm)	5	5
Weight comparison (threshold, g)	20	40
Graphesthesia (number from 0 to 9, /10)	10	1
Two‐point discrimination (tip of the middle finger, mm)	3	>25
Tactile localization (distances, mm)		
Forced pointing	<20	>50‐100
Three‐dimensional geometric figures		
Identification (max: 42)	42	0
Forced choice out of six visually presented figures (max: 42)^a^	42	18^b^
Materials		
Identification (max: 42)	42	0
Forced choice out of six visually presented materials (max: 42)^a^	42	16^c^
Everyday objects		
Identification (max: 30)	30	0
Forced choice out of six visually presented objects (max: 30)^a^	30	21^b^

Note

min = minimum. max = maximum.

^a^ Bayesian binomial test was conducted

^b^ Showed extreme evidence in favour of ‘above‐chance performance'

^c^ Showed strong evidence in favour of ‘above‐chance performance'

#### Cortical somatosensory abilities

Evaluations of cortical somatosensory modalities (Klingner & Witte, 2018) (Table 2B) showed that the patient had conscious perception of the size and weight of stimuli; thus, the ability to perform normal size comparisons was preserved, and that for performing weight comparison was also mostly preserved. However, all other tests revealed significant impairments. As in the other 'forced‐choice' tests, the patient also had a remarkably low number of correct answers in those assessing graphesthesia. In the two‐point discrimination test, the patient was unable to perceive being touched and to give correct responses, even if his fingertip was touched at a 25 mm distance. In the tactile localization test, the patient was unable to perceive being touched and unable to touch the touched point using his non‐impaired limb, with up to 50 mm between the touched point and the point where he touched. No improvement in performance was achieved with ‘forced‐choice’ tests.

The patient was unable to spontaneously identify three‐dimensional geometric figures or everyday objects based on his tactile sense or materials based on their texture, despite six options being provided. However, if the patient was forced to choose among them, responses were correct at a much higher frequency than expected by chance (geometric figures, 18/42 (correct rate 42.9%), BF_01_ = 0.002; materials, 16/42 (correct rate 38.1%), BF_01_ = 0.018; objects, 21/30 (correct rate 70.0%), BF_01_ < 0.001). Nevertheless, the patient complained that, no matter what was touched, 'everything was the same', and he 'could not even tell whether something was round or sharp'. In addition, the patient had no sense of whether he was giving correct responses. Even if he touched very different materials, such as files and aluminium, he reported ‘I can't tell if it's rough or slippery’. When the examiner asked, ‘Are metal and plastic different in touch?’, the patient replied, ‘I don't know at all’. In addition, the patient reported that when he was touching the key, he did not ‘have any feeling. I can't perceive the length at all. I have no clue… I don't have any feeling when I touch it’. The patient repeated these observations every time he was tested and had extremely low confidence. Hand movements to explore the three‐dimensional geometric figures and everyday objects, even when making the wrong selection, were smooth and adapted to the shape of the object.

### Somatosensory‐evoked potential

Cortical potentials were detected over both hemispheres after stimulation of the contralateral median nerves (Figure 1b). N20 was evoked at 18.1 msec with 1.39 µV in the right hemisphere and at 18.1 msec with 1.29 µV in the left. There was no difference in the latency and amplitude between hemispheres.

## Discussion

The patient was impaired in his perception of the stimuli of all primary somatosensory modalities except for vibration and all cortical somatosensory modalities except for weight and size. Regarding primary somatosensory modalities and tactile localization, responses were markedly impaired, even in cases of forced selection. Therefore, we concluded that the patient did not exhibit numbsense of these sensations. The patient could consciously perceive the weight and size stimuli; thus, his condition did not meet the definition of numbsense. However, when the patient was forced to choose among options, he performed considerably above‐chance in selecting three‐dimensional geometric figures, materials, and everyday objects that he touched. These results suggest that the patient exhibited numbsense regarding the characteristics of these objects. As described above, geometric figures weighed almost the same and had almost the same volume. Therefore, it would have been difficult for the patient to judge geometric shapes based on such a slight difference in size and weight. In the recognition test for texture, the patient was asked to touch the surface of a fixed material of the same size. Therefore, we think preserved sensation of weight and size was not available to judge the texture. However, it might be easier for the patient to narrow down the target items from the choices since his weight and size sensations were preserved. Thus, this may explain the better performance for everyday objects than that for texture and geometric shapes. Considering that forced localization did not improve the patient’s tactile localization, the numbsense observed here differs from traditional definition of the phenomenon and appears to be associated with a different mechanism.

As indicated by brain images and somatosensory‐evoked potentials, the patient’s postcentral gyrus lesion did not include the S1 region, which receives information from the upper limbs. This suggests that all somatosensory information concerning the upper limbs was transmitted to S1. Schröder, Schmidt, and Blankenburg (2019) conducted a functional magnetic resonance imaging study with electrical pulse stimulation and demonstrated that S2 activity was most relevant to the emergence of perceptual awareness of a stimulus. Therefore, S2 is thought to be essential for recognizing at least some types of somatic sensations. This suggests that many sensations could not be recognized by this patient because S2 was included in the lesion of the parietal operculum.

James, Kim, and Fisher (2007; James & Kim, 2010) proposed that there are two streams of somatosensory information processing after S1: dorsal and ventral. The dorsal stream receives information from deep receptors, such as muscle spindles, and merges with the visual dorsal stream at the intraparietal sulcus (IPS) without passing through S2. Information that reaches the intraparietal sulcus is used to control actions. There may have been no problems with hand movement when exploring objects because the patient had no lesion in the intraparietal sulcus. The sensation of vibration is transmitted from muscle spindles (Fallon & Macefield, 2007). Opening the hand to measure the size and weight induces tension in the muscle, which is an appropriate stimulation for the muscle spindle. Accordingly, the patient may have had minimal problems with these sensations because he had no lesion in the dorsal stream.

Blindsight, which is equivalent to numbsense in vision, does not occur in all patients with brain damage to areas such as the primary visual cortex (Weiskrantz, 1986). Similarly, we do not think that tactile localization numbsense would occur in all patients, although we do not know what factors determine the numbsense of tactile localization. Blindsight can be divided into the route via pulvinar and the route via interlaminar layers of the lateral geniculate body (Danckert and Rossetti, 2005). In the former route, blindsight may occur in the detection of an achromatic stimulus (Stoerig, Hübner, & Pöppel, 1985), while in the latter route, blindsight may occur in the detection of a chromatic stimulus but may not occur in the detection of an achromatic stimulus (Stoerig, 1987). We consider that a similar dissociation occurred in this patient, wherein numbsense of shape, texture, and objects occurred but numbsense of tactile localization did not occur.

The tactile ventral stream receives information from receptors distributed in the skin and merges with the visual ventral stream at the lateral occipital complex through S2. Information reaching the lateral occipital complex is used to recognize the shape of an object (James & Kim, 2010). Regarding the pathway used for texture recognition, functional magnetic resonance imaging research has shown that the tactile ventral stream may merge with the visual ventral stream at the right medial occipital cortex through S2 (Stilla & Sathian, 2008). Shape and texture information is important for the identification of everyday objects (Reed, Caselli & Ferah, 1996; Bohlhalter, Fretz & Weber, 2002). Our patient performed considerably above‐chance while selecting the touched shapes, textures, and everyday objects, which suggests that shape and texture information was transmitted to the lateral occipital complex and the right medial occipital cortex through another ventral pathway that does not pass through S2, allowing for collation without the possibility of tactile awareness.

In conclusion, our findings in this case strengthen the hypothesis that there are separate dorsal and ventral streams of somatosensory information processing after S1. Furthermore, the proposal of a covert recognition system may help to guide the conceptual framework regarding the mechanisms of somatosensory perception and object recognition; further studies are warranted to explore these findings.

## Conflict of interest

All authors declare no conflict of interest.

## Author contributions

Keisuke Hanada, MS (Conceptualization; Data curation; Investigation; Writing – original draft; Writing – review & editing) Kayoko Yokoi (Writing – original draft; Writing – review & editing) Akinori Futamura (Visualization; Writing – review & editing) Yuji Kinoshita (Data curation; Investigation) Kazutaka Sakamoto (Writing – original draft; Writing – review & editing) Kenjiro Ono (Supervision; Visualization) Kazumi Hirayama (Conceptualization; Project administration; Supervision; Writing – original draft; Writing – review & editing).

## Data Availability

The data are not available for public access because of patient privacy concerns, but are available from the corresponding author on reasonable request.
